# Ontogenetic variation in the diet of the anuran community from a semi-arid environment in the southeastern Chihuahuan Desert

**DOI:** 10.7717/peerj.7908

**Published:** 2019-10-18

**Authors:** Ricardo Luría-Manzano, Aurelio Ramírez-Bautista

**Affiliations:** Laboratorio de Ecología de Poblaciones, Centro de Investigación Biológica, Instituto de Ciencias Básicas e Ingeniería, Universidad Autónoma del Estado de Hidalgo, Pachuca, Hidalgo, México

**Keywords:** Dietary composition, Formicidae, Diptera, Intraspecific variation, Trophic niche width

## Abstract

Although ontogeny influences dietary composition and trophic niche breadth in many anurans, its effects on diet have been little analyzed in sympatric species. In this study, we analyzed interspecific and ontogenetic variation in dietary composition and trophic niche width in an anuran community from a semi-arid environment. We found a more profound effect of species identity than body size on dietary composition, with the diet of four species dominated by formicids, that of two others by coleopterans and formicids, and that of the remaining species not dominated by specific prey types. We found ontogenetic changes in dietary composition in three of four species analyzed, in which consumption of some small insects decreased as predator size increased, regardless of species. Additionally, we did not find ontogenetic change in prey number consumed in any of the four species, but prey size increased with increasing predator size in all of them. Most species exhibited a narrow trophic niche, which was even narrower in adults in three of the four species analyzed. Costello’s modified plots revealed a high variation among individuals in termite consumption in *Anaxyrus punctatus*, and in more prey types in *Spea multiplicata*. Our results suggest that this community is not size-structured, and that ontogenetic diet shifts are mainly caused by passive sampling toward prey of different sizes. Finally, comparisons with previous data revealed an interpopulation pattern, in which trophic niche width contracts as aridity increases, possibly because of an increase in interspecific competition for trophic resources.

## Introduction

Anurans exhibit a great variation in dietary composition and trophic niche breadth, with some of them able to select prey to a certain degree (Toft, 1980; Flowers & Graves, 1995; Cogălniceanu, Palmer & Ciubuc, 2000). This variation is evident in sympatric species, with few of them located at either of the extremes from a specialized to a generalized diet, and most occupying an intermediate position along this continuum (Toft, 1980; Vitt & Caldwell, 1994; Caldwell & Vitt, 1999).

In such conditions of sympatry, characteristics of individuals potentially become important determinants of dietary aspects, both within and between species. Among these characteristics, it has been found in numerous single-species studies that ontogeny influences both dietary composition (Donnelly, 1991; Lima & Moreira, 1993; Hirai, 2002; Biavati, Wiederhecker & Colli, 2004; Valderrama-Vernaza, Ramírez-Pinilla & Serrano-Cardozo, 2009) and trophic niche breadth (Christian, 1982; Quiroga, Sanabria & Acosta, 2009; Ngo, Lee & Ngo, 2014). Nevertheless, the effects of this factor in sympatric anuran species have been little analyzed. For instance, Lima (1998) found that ontogenetic shifts in dietary composition are pervasive in six species of litter anurans from Central Amazonia, and Whitfield & Donnelly (2006) found that this phenomenon is present in half of the species from La Selva, Costa Rica. Ontogenetic shifts are relevant for sympatric species, because these can influence the outcome of interspecific interactions (such as competition and predation) and structure of the community (Werner & Gilliam, 1984; Polis, 1991).

The aforementioned studies on anuran communities and assemblages have been carried out in tropical ecosystems, mainly wet forests at low elevations; whereas drier habitats such as xeric regions of the world have received much less attention. Most anurans in the latter environments have to restrict their reproductive activities to the short rainy season (Creusere & Whitford, 1976; Torres-Cervantes et al., 2019), which for those species that hibernate under ground (e.g., scaphiopodids) or water (e.g., some ranids) potentially becomes the only period in which they can feed (Wells, 2007). Additionally, anurans inhabiting these environments must obtain the required energy reserves to survive, grow, and breed from species-poor arthropod communities, mainly dominated by ants and termites (MacKay, 1991; Rojas & Fragoso, 2000). Available data on dietary aspects of anurans from xeric regions show that members of Bufonidae and Scaphiopodidae (characteristic families of these environments) rely heavily on beetles, ants, and/or termites, and have relatively narrow trophic niches (Anderson, Haukos & Anderson, 1999; Newman, 1999; Quiroga, Sanabria & Acosta, 2009; Smith et al., 2011; Sulieman, Pengsakul & Afifi, 2016).

The underrepresentation of dietary studies on anuran communities in xeric environments is not surprising, considering that only ~7% of the anuran species of the world inhabit such environments (Warburg, 1997). Among these, the Chihuahuan Desert, located in southern United States and northern Mexico, is one of the most biologically diverse xeric regions in the world (Fitzgerald et al., 2004), with 22 anuran species inhabiting the Mexican portion (Lavín-Murcio & Lazcano, 2010; Cruz-Elizalde et al., 2016).

In this study, we analyze ontogenetic shifts in diet of the pond-breeding anuran community in a semi-arid region from the Chihuahuan Desert, Mexico. This community is composed of seven species in four families: Bufonidae, Hylidae, Ranidae, and Scaphiopodidae, and all of them are active and breed in the rainy season (Torres-Cervantes et al., 2019). We particularly address the following questions: (1) How do species differ in dietary composition? (2) Do ontogenetic shifts in dietary composition, and prey number and size exist in any of these species? and (3) Do ontogenetic changes in trophic niche width and feeding strategy exist in any of them? If this community is size-structured, we then expect that ontogeny (anuran size) will have as profound an effect on dietary composition as species identity. Moreover, if this structure is generated mainly through passive sampling of trophic resources (sensu Whitfield & Donnelly, 2006), we then expect that ontogenetic changes in prey size will be widespread, and that consumption of each prey type will change in the same direction (increase or decrease) as anuran size, regardless of the species. Finally, we discuss the likely effects that semi-aridity exerts on dietary aspects of some of the species studied here, in light of literature available regarding the diet of some of them from environments with different precipitation regimes (Castañeda-Gaytán et al., 2006; Smith et al., 2011; Hernández-Salinas et al., 2018).

## Methods

### Study site and collection of specimens

Individuals examined in this study were collected for a study of reproduction (Torres-Cervantes et al., 2019), under scientific permit SGPA/DGVS/01920/11 provided by Secretaría de Medio Ambiente y Recursos Naturales (SEMARNAT), and remain deposited in the herpetological collection of the Centro de Investigaciones Biológicas (CIB) of the Universidad Autónoma del Estado de Hidalgo (UAEH). They were found mainly from 19:00 to 24:00 h, in the immediacy of water bodies or in them, during the rainy seasons of four consecutive years (1996–1999). The study area covers five localities (maximum distance between them: ~38 km) within the municipality of Guadalcázar, San Luis Potosí, Mexico, in the southeastern portion of the Chihuahuan Desert (22°33′7″–22°53′31″N, 100°4′48″–100°26′44″W; from 1,086 to 1,365 m asl; Fig. S1). The vegetation type in these semi-arid localities is microphyll or rosette xeric scrub, with a mean annual temperature of 22.1 °C (range 16.4–25.9 °C; Fig. S2). The precipitation is seasonal, and hence there is a distinct dry season (October to April, 90.6 mm rainfall) and rainy season (May to September, 262.2 mm rainfall, Fig. S2; Comisión Nacional del Agua-Dirección General de Estudios (CONAGUA-DGE, 2018)). The amphibian fauna of the municipality of Guadalcázar comprises a total of nine anuran species, whose richness is concentrated in the arid and semi-arid environments (Cruz-Elizalde et al., 2016).

### Sample analysis

We measured snout–vent length (SVL) of anurans with a digital caliper (±0.01 mm), and assigned them to one of two size classes: small (11.47–48.65 mm SVL) or large (48.66–85.83 mm SVL). We determined sexual maturity of male individuals by the presence of nuptial excrescences and dark coloration of the throat (in the four species of bufonids), or only by the presence of one of these characteristics (nuptial excrescences in *Lithobates berlandieri* and *Spea multiplicata*, dark coloration of the throat in *Dryophytes eximius*). In females, the presence of ovarian follicles and convoluted oviducts were used for this purpose in all species. For each specimen, we removed both the stomach and gut contents as recommended by Schoener (1989) to increase the sample size. Contents were sorted in Petri dishes, and prey items counted, measured, and identified taxonomically at order level. Additionally, we separated formicids from other hymenopterans, and larvae from adults in holometabolous insects (Whitfield & Donnelly, 2006). We measured length and width of each prey item with a digital caliper (±0.01 mm), and estimated its volume with the formula for a prolate spheroid (Caldwell & Vitt, 1999). Additionally, we recorded the presence of plant matter to compute its frequency of occurrence (FO); however, it was not included in further diet analyses (Cogălniceanu, Palmer & Ciubuc, 2000).

### Data analyses

Preliminary analyses showed that dietary composition did not differ by year in any species (*P* > 0.08 in all cases) and that maximum difference of intraspecific trophic width among years was 0.141. Therefore, we pooled data from the 4 years for all analyses. To analyze interspecific and ontogenetic (i.e., body size) variation in dietary composition, we constructed dissimilarity matrices with the numeric and volumetric proportions of prey categories eaten by each individual, using the Bray–Curtis dissimilarity index. These matrices were the basis of the following three analyses. First, we conducted nonmetric multidimensional scaling (nMDS) to depict variation in diet composition among species and size classes. Subsequently, we included these two factors in two-way analyses of similarities (ANOSIM; Clarke, 1993) to test for significant differences between them (Clarke, 1993). Lastly, we conducted similarity percentage analyses (SIMPER; Clarke, 1993; Clarke & Warwick, 2001) between those groups that differed significantly in diet composition to determine prey categories that contributed most to these differences. The nMDS were conducted considering the seven species of the community, but the ANOSIMs and SIMPERs only with the four species for which we had non-adult and adult specimens (see “Results”).

Analysis of similarities tests for an overall effect of predator size on dietary composition regardless of species, but it does not test this effect within each of them. To evaluate ontogenetic shifts in dietary composition for each species, we first computed two matrices. The first included the absolute values of the difference in SVL between each pair of individuals, and the second included differences in diet calculated with the Bray–Curtis dissimilarity index (Whitfield & Donnelly, 2006). If there were ontogenetic diet shifts, we would expect that more dissimilar-sized individuals have more dissimilar diets, and therefore the two matrices would be positively correlated. We tested for this correlation with a simple Mantel test with 1,000 permutations (Whitfield & Donnelly, 2006; Araújo et al., 2007). We calculated Spearman’s correlations between SVL of predators and proportion of prey types that had a FO ≥ 30% in any of the age classes to detect prey whose consumption changed with SVL, in those species that showed ontogenetic shifts according to the Mantel test. To test for ontogenetic change in prey number and size (volume) in each species, we calculated Pearson correlations between each of these variables and predator SVL. We transformed variables to their natural log when necessary to meet assumptions of normality (Zar, 2010).

We calculated trophic niche width for each size class and species with Levin’s standardized index (Hurlbert, 1978), with both the numeric and volumetric proportions of prey categories consumed. We did the same for three studies (Castañeda-Gaytán et al., 2006; Smith et al., 2011; Hernández-Salinas et al., 2018) which did not report trophic niche width, to detect possible geographical variations in this dietary variable. We represented the feeding strategy of each size class and species graphically with the Costello method (Costello, 1990) modified by Amundsen, Gabler & Staldvik (1996). This technique positions all the prey types consumed on a two-dimensional graph, with FO on the *x*-axis and prey-specific abundance on the *y*-axis. Prey-specific abundance (*P*_*i*_) was calculated as the proportion (in terms of number or volume) of the prey type (*i*) in relation to the total prey items, considering only those individuals in which the prey (*i*) is found. The graph provides information about three aspects of the diet: prey importance, niche width of the sample (from generalization to specialization), and contribution of individuals to the niche width (from between- to within-phenotype contributions) (Amundsen, Gabler & Staldvik, 1996). The nMDS, ANOSIM, and SIMPER analyses were performed in PRIMER 7.0.13 (Clarke & Gorley, 2015), the Mantel tests in PAST (Hammer, Harper & Ryan, 2001), and the remaining analyses in NCSS 10. Means are shown ± 1 SD.

## Results

We analyzed 359 individuals, of which 289 had gastrointestinal prey remnants (Table 1). We had samples of both non-adults and adults for the following four species: *Anaxyrus punctatus*, *D. eximius*, *L. berlandieri*, and *S. multiplicata*. Size of specimens ranged from a young *S. multiplicata* with a SVL = 11.47 mm to an adult *Incilius nebulifer* with a SVL = 85.83 mm (Table 1). From the samples that yielded prey remnants, we identified 8,689 prey items, representing 26 prey categories (Table 2).

**Table 1:** Sample and body sizes for each species. Number of individuals by age class and presence of each type of matter (with percentage in parentheses), and snout–vent length (SVL) (with range in parentheses) of the anuran community at the study site. Plant matter percentages shown are for individuals with non-empty stomachs.

Species	Number of individuals					Mean SVL (mm)	
	Non-adults	Adults	Total	Prey remnants (%)	Plant matter (%)	Non-adult individuals	Adult individuals
*Anaxyrus cognatus*	0	10	10	8 (80)	4 (50)	–	68.87 ± 12.65 (54.03–83.18)
*Anaxyrus debilis*	0	11	11	10 (90.91)	4 (40)	–	39.98 ± 4.18 (33.08–45.68)
*Anaxyrus punctatus*	33	50	83	78 (93.98)	22 (28.21)	30.11 ± 9.29 (12.99–48.55)	55.88 ± 5.17 (49.06–70.92)
*Dryophytes eximius*	11	33	44	32 (72.73)	3 (9.38)	18.42 ± 2.7 (15.45–24.89)	29.8 ± 2.75 (24.09–36.62)
*Incilius nebulifer*	0	21	21	20 (95.24)	12 (60)	–	69.85 ± 7.1 (54.83–85.83)
*Lithobates berlandieri*	14	85	99	80 (80.81)	19 (23.75)	40.53 ± 5.1 (33.03–46.87)	60.94 ± 8.14 (48.73–84.41)
*Spea multiplicata*	63	28	91	61 (67.03)	6 (9.84)	20.3 ± 8.82 (11.47–47.81)	53.35 ± 2.66 (49.28–58.17)

**Table 2:** Diet of the seven species of anurans. Dietary composition and trophic niche width of the seven species of anurans analyzed herein. Total number (*N*) and volume (mm^3^; *V*) of each prey category, with percentages in parentheses. Main prey categories whose accumulated percentage made up at least 70% of the total prey consumed are in bold.

Prey category	*Anaxyrus cognatus*		*Anaxyrus debilis*		*Anaxyrus punctatus*				*Dryophytes eximius*			
	Large (adults)		Small (adults)		Small (non-adults)		Large (adults)		Small (non-adults)		Small (adults)	
	*N* (%)	*V* (%)	*N* (%)	*V* (%)	*N* (%)	*V* (%)	*N* (%)	*V* (%)	*N* (%)	*V* (%)	*N* (%)	*V* (%)
Arachnida												
Acari	0	0	0	0	5 (0.3)	1.17 (0.04)	0	0	0	0	0	0
Araneae	4 (0.63)	20.58 (0.15)	0	0	10 (0.6)	3.6 (0.13)	5 (0.11)	44.43 (0.29)	**14 (16.87)**	8.07 (8.83)	4 (3.15)	31.83 (3.52)
Scorpiones	1 (0.16)	30.84 (0.23)	0	0	0	0	1 (0.02)	15.48 (0.1)	0	0	0	0
Solifugae	0	0	0	0	0	0	0	0	0	0	0	0
Crustacea												
Isopoda	0	0	0	0	0	0	1 (0.02)	86.68 (0.56)	0	0	0	0
Insecta												
Coleoptera (A)	39 (6.15)	**4334.35 (32.52)**	3 (0.7)	37.96 (11.88)	88 (5.31)	420.89 (14.61)	40 (0.86)	769.59 (5.01)	**12 (14.46)**	**40.65 (44.49)**	**49 (38.58)**	**363.74 (40.23)**
Coleoptera (L)	1 (0.16)	22.9 (0.17)	0	0	0	0	0	0	2 (2.41)	2.08 (2.28)	0	0
Collembola	0	0	0	0	**409 (24.7)**	97.18 (3.37)	0	0	4 (4.82)	0.07 (0.08)	0	0
Dermaptera	0	0	1 (0.23)	0.05 (0.02)	0	1.21 (0.04)	2 (0.04)	15.99 (0.1)	0	0	0	0
Diptera (A)	4 (0.63)	1.71 (0.01)	0	0	41 (2.48)	63.24 (2.2)	2 (0.04)	24.96 (0.16)	**21 (25.3)**	8.86 (9.69)	12 (9.45)	17.77 (1.97)
Diptera (L)	0	0	0	0	0	0	0	0	1 (1.2)	2.54 (2.78)	0	0
Ephemeroptera	0	0	0	0	0	0	0	0	0	0	0	0
Hemiptera	3 (0.47)	68.6 (0.51)	0	0	6 (0.36)	8.2 (0.28)	10 (0.21)	123.44 (0.8)	1 (1.2)	2.5 (2.73)	7 (5.51)	**196.04 (21.68)**
Homoptera	1 (0.16)	3.9 (0.03)	0	0	21 (1.27)	17.1 (0.59)	1 (0.02)	3.29 (0.02)	6 (7.23)	**11.51 (12.6)**	1 (0.79)	1.23 (0.14)
Hymenoptera (Formicidae)	**506 (79.81)**	**7710.58 (57.85)**	**160 (37.12)**	**99.16 (31.05)**	**919 (55.5)**	**1517.05 (52.67)**	**2685 (57.42)**	**10640.97 (69.25)**	**18 (21.69)**	**12.07 (13.21)**	**45 (35.43)**	77.33 (8.55)
Hymenoptera (others)	4 (0.63)	38 (0.29)	0	0	2 (0.12)	1.58 (0.05)	5 (0.11)	15.2 (0.1)	2 (2.41)	2.47 (2.71)	3 (2.36)	**186.64 (20.64)**
Isoptera	58 (9.15)	52.95 (0.4)	**266 (61.72)**	**180.57 (56.54)**	139 (8.39)	**625.95 (21.73)**	**1894 (40.5)**	1739.87 (11.32)	0	0	0	0
Lepidoptera (A)	1 (0.16)	12.65 (0.09)	0	0	5 (0.3)	55.46 (1.93)	4 (0.09)	152.76 (0.99)	0	0	2 (1.57)	10.6 (1.17)
Lepidoptera (L)	2 (0.32)	198.46 (1.49)	1 (0.23)	1.64 (0.51)	10 (0.6)	65.79 (2.28)	22 (0.47)	1677.89 (10.92)	0	0	3 (2.36)	7.99 (0.88)
Neuroptera (A)	0	0	0	0	0	0	0	0	0	0	0	0
Odonata	7 (1.1)	628.21 (4.71)	0	0	0	0	0	0	0	0	0	0
Orthoptera	3 (0.47)	205.73 (1.54)	0	0	1 (0.06)	1.61 (0.06)	3 (0.06)	50.92 (0.33)	2 (2.41)	0.54 (0.59)	1 (0.79)	11.02 (1.22)
Psocoptera	0	0	0	0	0	0	0	0	0	0	0	0
Myriapoda												
Diplopoda	0	0	0	0	0	0	0	0	0	0	0	0
Vertebrata												
Anura	0	0	0	0	0	0	0	0	0	0	0	0
Aves	0	0	0	0	0	0	1 (0.02)	3.6 (0.02)	0	0	0	0
Total	634 (100)	13329.46 (100)	431 (100)	319.37 (100)	1656 (100)	2880.02 (100)	4676 (100)	15365.06 (100)	83 (100)	91.37 (100)	127 (100)	904.2 (100)
Trophic niche width	0.04	0.1	0.23	0.33	0.14	0.16	0.07	0.07	0.49	0.3	0.27	0.32

Prey category	*Incilius nebulifer*		*Lithobates berlandieri*				*Spea multiplicata*			
	Large (adults)		Small (non-adults)		Large (adults)		Small (non-adults)		Large (adults)	
	*N* (%)	*V* (%)	*N* (%)	*V* (%)	*N* (%)	*V* (%)	*N* (%)	*V* (%)	*N* (%)	*V* (%)
Arachnida										
Acari	0	0	0	0	0	0	25 (6.19)	1.71 (0.3)	0	0
Araneae	4 (1.6)	9.8 (0.07)	1 (1.39)	0.53 (0.1)	14 (4.79)	1654.69 (8.68)	7 (1.73)	9.07 (1.6)	2 (3.13)	**91.86 (10.33)**
Scorpiones	0	0	0	0	0	0	0	0	0	0
Solifugae	0	0	0	0	0	0	0	0	1 (1.56)	**73.65 (8.28)**
Crustacea										
Isopoda	0	0	0	0	3 (1.03)	405.37 (2.13)	0	0	0	0
Insecta										
Coleoptera (A)	**62 (24.8)**	**10536.64 (79.1)**	**15 (20.83)**	**124.86 (23.81)**	**62 (21.23)**	**3154.27 (16.55)**	**96 (23.76)**	**130.43 (22.99)**	**6 (9.38)**	47.6 (5.35)
Coleoptera (L)	0	0	**7 (9.72)**	73.78 (14.07)	15 (5.14)	175.21 (0.92)	6 (1.49)	11.78 (2.08)	3 (4.69)	66.61 (7.49)
Collembola	0	0	1 (1.39)	0.02 (<0.01)	0	0	3 (0.74)	0.38 (0.07)	0	0
Dermaptera	0	0	2 (2.78)	**77.01 (14.69)**	2 (0.68)	128.17 (0.67)	2 (0.5)	0.56 (0.1)	2 (3.13)	**144.46 (16.24)**
Diptera (A)	1 (0.4)	0.36 (<0.01)	**21 (29.17)**	23.17 (4.42)	**28 (9.59)**	193.88 (1.02)	7 (1.73)	4.3 (0.76)	**4 (6.25)**	1.78 (0.2)
Diptera (L)	0	0	0	0	0	0	1 (0.25)	10.74 (1.89)	0	0
Ephemeroptera	0	0	2 (2.78)	9.12 (1.74)	0	0	0	0	0	0
Hemiptera	22 (8.8)	54.96 (0.41)	4 (5.56)	9.8 (1.87)	13 (4.45)	1358.71 (7.13)	3 (0.74)	2.92 (0.51)	0	0
Homoptera	0	0	4 (5.56)	10.1 (1.93)	3 (1.03)	46.33 (0.24)	14 (3.47)	24.94 (4.4)	1 (1.56)	1.09 (0.12)
Hymenoptera (Formicidae)	**125 (50)**	494.34 (3.71)	4 (5.56)	13.89 (2.65)	**25 (8.56)**	217.84 (1.14)	**138 (34.16)**	**127.1 (22.4)**	**36 (56.25)**	45.85 (5.15)
Hymenoptera (others)	1 (0.4)	42.67 (0.32)	**5 (6.94)**	8.53 (1.63)	24 (8.22)	1134.1 (5.95)	20 (4.95)	6.33 (1.12)	0	0
Isoptera	20 (8)	12.25 (0.09)	0	0	0	0	**70 (17.33)**	**70.34 (12.4)**	2 (3.13)	0.7 (0.08)
Lepidoptera (A)	1 (0.4)	7.69 (0.06)	**5 (6.94)**	**159.34 (30.39)**	**64 (21.92)**	**4835.85 (25.37)**	1 (0.25)	25.34 (4.47)	1 (1.56)	0.48 (0.05)
Lepidoptera (L)	0	0	0	0	12 (4.11)	792.86 (4.16)	7 (1.73)	59.13 (10.42)	2 (3.13)	29.33 (3.3)
Neuroptera (A)	2 (0.8)	8.61 (0.06)	0	0	0	0	0	0	0	0
Odonata	0	0	0	0	1 (0.34)	15.65 (0.08)	0	0	0	0
Orthoptera	11 (4.4)	2117.88 (15.9)	1 (1.39)	14.18 (2.7)	**25 (8.56)**	**4940.76 (25.92)**	3 (0.74)	**82.1 (14.47)**	3 (4.69)	**329.8 (37.08)**
Psocoptera	0	0	0	0	0	0	1 (0.25)	0.17 (0.03)	0	0
Myriapoda										
Diplopoda	1 (0.4)	34.95 (0.26)	0	0	0	0	0	0	0	0
Vertebrata										
Anura	0	0	0	0	0	0	0	0	1 (1.56)	56.32 (6.33)
Aves	0	0	0	0	1 (0.34)	6.04 (0.03)	0	0	0	0
Total	250 (100)	13320.18 (100)	72 (100)	524.33 (100)	292 (100)	19059.71 (100)	404 (100)	567.33 (100)	64 (100)	889.53 (100)
Trophic niche width	0.2	0.05	0.44	0.34	0.47	0.33	0.23	0.34	0.16	0.34

**Note:**

A, Adults; L = Larvae.

### Dietary composition

The diet of all the species was composed entirely or mainly by invertebrates; we found consumption of vertebrates in only three of them: we found much digested feathers, one in each *A. punctatus* and *L. berlandieri*, and unidentifiable remains of one postmetamoprhic anuran in an adult *S. multiplicata*. Formicidae, or Formicidae and Coleoptera (adults), in various degrees numerically dominated the diet of most species in the community. The exception was *L. berlandieri*, in which Coleoptera (adults), Lepidoptera (adults), and Diptera (adults) dominated. In terms of volume, Formicidae and/or Coleoptera (adults) overwhelmingly comprised the bulk of the diet of the four bufonids, whereas no particular prey type predominated in the diet of the other three species. Nevertheless, Coleoptera (adults) and Hemiptera were the main prey categories in the diet of *D. eximius*, whereas Lepidoptera (adults) and Orthoptera were the main categories for *L. berlandieri*, and Orthoptera and Coleoptera (adult) for *S. multiplicata* (Table 2). Consumption of plant matter varied greatly among species, from the lowest FO of 9.38% in *D. eximius*, to the highest of 60% in *I. nebulifer*. Consumption of inorganic matter was low in the whole community, from a FO of 3.13% in *D. eximius*, to 25% in *A. cognatus* (Table 1).

**Figure 1:**
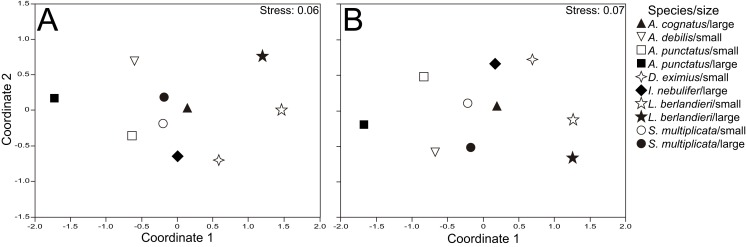
Nonmetric multidimensional scaling for dietary composition. Nonmetric multidimensional scaling ordinations showing variation in diet between size classes and species, for numeric (A) and volumetric proportions (B) of prey categories consumed.

The nMDS plots showed that samples of different size classes within species tended to cluster more than samples of different species within size classes, for numeric (Fig. 1A) and volumetric (Fig. 1B) proportions of prey types consumed. This was supported by the two-way ANOSIMs, which indicated significant differences in diet composition between body sizes (Global *R* = 0.075, *P* = 0.034 for number; Global *R* = 0.072, *P* = 0.044 for volume) but even more profound differences between species (Global *R* = 0.271, *P* = 0.001 for both number and volume). Pairwise comparisons with ANOSIM and SIMPER results showed significant differences among almost all species, with Formicidae and Coleoptera (adults) contributing most to these differences (Table S1). *Anaxyrus punctatus* consumed more formicids than *D. eximius* and *S. multiplicata*, and these in turn more than *L. berlandieri*. Moreover, *D. eximius* and *S. multiplicata* ate more coleopterans than *A. punctatus* and *L. berlandieri* (Table S1).

We were able to test for ontogenetic diet shifts in the four species for which we had both non-adults and adults. We found ontogenetic change in diet composition in all of them with the exception of *S. multiplicata* (Table 3). We found some similarities in species that showed this dietary change, such as a decrease in consumption of adult dipterans (in the three species) and homopterans (in two species) with increasing SVL. Consumption of adult coleopterans also changed with predator size in the three species; whereas it decreased with increasing SVL in *A. punctatus* and *L. berlandieri*, it increased with increasing SVL in *D. eximius* (Table S2). Consumption of formicids did not change in relation to SVL in any species. We did not find ontogenetic change in prey number consumed by any species, but prey size changed with SVL, increasing with increasing predator size in the four species analyzed (Table 3).

**Table 3:** Correlations between anuran size and dietary variables. Results of correlations that tested changes in dietary composition, and prey number and size in relation to anuran size, in those species for which we had both non-adults and adults.

	Dietary composition				Prey number		Prey size	
	Number		Volume		*R*	*P*	*R*	*P*
	*R*	*P*	*R*	*P*				
*Anaxyrus punctatus*	0.17	0.007	0.11	0.03	0.1	0.37	0.57	<0.0001
*Dryophytes eximius*	0.24	0.002	0.25	<0.0001	−0.25	0.16	0.67	0.0009
*Lithobates berlandieri*	0.11	0.01	0.13	0.004	0.04	0.75	0.42	0.004
*Spea multiplicata*	0.03	0.21	0.08	0.06	−0.05	0.73	0.36	0.04

### Trophic niche width and feeding strategy

Trophic niche width varied from 0.04 in adults of *A. cognatus* to 0.49 in non-adults of *D. eximius*. The four bufonids and *S. multiplicata* showed a narrow trophic niche, and the remaining two species an intermediate one (Table 2). Intraspecifically, trophic niche for numeric proportions was wider in non-adults than in adults in *A. punctatus*, *D. eximius*, and *S. multiplicata*; but it was similar between age classes in *L. berlandieri*. Volumetric trophic niche was only wider in non-adults than in adults in *A. punctatus* (Table 2).

Costello’s modified plots represented the position of species along the continuum from specialized to generalist diet, and highlighted between and within phenotype contributions (i.e., variation in resource use among individuals) to their trophic niche width. *Anaxyrus cognatus*, *A. debilis*, *A. punctatus*, and *S. multiplicata* relied heavily on formicids, whereas *I. nebulifer* relied on coleopterans and formicids. Moreover, the position of Isoptera toward the upper left corner of the graph in *A. punctatus* revealed a high variation among individuals in consumption of this prey. The position of more prey categories toward the same part of the graph in *S. multiplicata* suggests a high between-phenotype component in food use in this species. On the other hand, *D. eximius* and *L. berlandieri* showed a more generalist pattern (many prey categories with *P_i_* < 0.5) with a relatively higher within than between-phenotype component. We did not observe evidence of ontogenetic shift in feeding strategy in the plots in any species of the community (Fig. S3; Salvidio et al., 2012).

## Discussion

A few studies have been carried out on dietary aspects of anuran communities from arid and semi-arid regions (Anderson, Haukos & Anderson, 1999; Hardy & Crnkovic, 2006). However, none of these has evaluated ontogenetic changes in dietary composition. Therefore, this is the first study that analyzes ontogenetic shifts in an anuran community from a semi-arid environment, even though we were not able to analyze these shifts in all the studied species. We were able to identify that: (1) species identity had a significant effect on dietary composition, (2) predator size had a less profound effect on prey types consumed, but three of the four species analyzed did change diet, and the four changed prey size with ontogeny, and (3) most species showed a narrow trophic niche, which was even narrower in adults of some of them.

Most researchers that have analyzed ontogenetic changes in dietary composition in anurans have found such changes (Lima, 1998; Hirai, 2002; Hodgkison & Hero, 2003). This suggests that it is a geographically and taxonomically widespread phenomenon, and pervasive among species of different sizes and foraging modes. We particularly found a decrease in consumption of small arthropods such as collembolans and dipterans as anuran size increased, which has been found elsewhere (Lima & Moreira, 1993; Blackburn & Moreau, 2006; Maragno & De Souza, 2011 for collembolans; Hirai, 2002; Hodgkison & Hero, 2003 for dipterans). Nevertheless, to our knowledge, the increase in consumption of adult lepidopterans as predator size increases (which we found in *L. berlandieri*) had only been reported in *Quasipaa verrucospinosa* (Ngo, Lee & Ngo, 2014), and our finding of decrease in consumption of homopterans with increasing SVL in two species has not been previously reported.

On the other hand, studies of small anuran species have found that as individuals grow they increase their consumption of ants (Donnelly, 1991; Valderrama-Vernaza, Ramírez-Pinilla & Serrano-Cardozo, 2009), or did not change it at all (Biavati, Wiederhecker & Colli, 2004; Maragno & De Souza, 2011). Conversely, Hirai (2002) found that consumption of ants decreased as frog size increased in a large ranid, *Pelophylax nigromaculatus*. Interestingly, none of the two ant-specialized toads for which we had non-adults and adults (*A. punctatus* and *S. multiplicata*) changed their consumption of this prey type with ontogeny (as also seen in Costello’s modified plots), in spite of their relatively large body size. The predominance of ants throughout the postmetamorphic size range in these two species is likely due to their high abundance and diversity in the Chihuahuan Desert (Rojas & Fragoso, 2000), and their often clustered distribution (Hardy & Crnkovic, 2006; Quiroga, Sanabria & Acosta, 2009).

We did not find evidence that the community was size-structured along the trophic niche, since species identity had a more profound effect than body size on diet, similar to that found in a leaf-litter herpetofaunal assemblage from Costa Rica (Whitfield & Donnelly, 2006). However, the pervasiveness of ontogenetic change in prey types and sizes in the anurans we studied allows us to suggest the likely mechanisms behind this phenomenon. The four species we analyzed consumed larger prey as they grew, and three of them showed ontogenetic shifts in dietary composition, in which consumption of small insects (e.g., dipterans, homopterans) decreased as their body size increased, regardless of the species. These results suggest that ontogenetic diet shifts are mainly caused by passive sampling toward prey of different sizes, an attribute that actually differs greatly among arthropod orders (Schoener & Janzen, 1968). Although this mechanism has been previously suggested (Labanick, 1976; Donnelly, 1991), ontogenetic changes in foraging activity (Lima & Magnusson, 2000) and electivity for prey types independent of prey size (Lima & Moreira, 1993; Lima, 1998) can also account for ontogenetic diet shifts. Foraging activity did not change with ontogeny in any of the species analyzed herein, as indicated by the constant number of prey they ingested throughout their body size range. On the other hand, narrower trophic niches in adults than in non-adults in *A. punctatus* and *D. eximius* may indicate that electivity for prey type changes ontogenetically in these species, with adults becoming more selective (Christian, 1982), which could account for our findings. Nevertheless, further research that analyzes prey availability in the environment is needed to test this hypothesis.

In our study site, the only species that did not change prey type consumption ontogenetically (i.e., *S. multiplicata*) was neither the one that showed the lowest range in body size nor the narrowest trophic niche, which is similar to what was found by Whitfield & Donnelly (2006). Moreover, numerous studies have found ontogenetic shifts in dietary composition in small anuran species (Donnelly, 1991; Lima & Moreira, 1993; Lima, 1998; Valderrama-Vernaza, Ramírez-Pinilla & Serrano-Cardozo, 2009). Small body size and narrow trophic niche therefore do not seem to be related to the non-existence of ontogenetic change in prey types consumed by anurans. Since the non-existence of this phenomenon seems to be uncommon in this vertebrate group, it is difficult to elucidate the factors associated with it. However, Araújo et al. (2007, 2009) did not find evidence of this phenomenon in eight anuran species from the Cerrado biome of Brazil, yet all of them exhibited diet variation not attributable to age (individual specialization; Bolnick et al., 2003), which also could be true for *S. multiplicata* from Guadalcázar, as shown in the modified Costello’s plots. Considering these results, we suggest that individual specialization may be related to the non-existence of ontogenetic diet change, through inter-individual variation within age classes exceeding variation between age classes. However, stable isotope analyses of the anuran species from Guadalcázar are needed to confirm this hypothesis.

Comparisons with available data from the rainy season and similar age classes for some of the species we analyzed enables us to understand the effects that semi-aridity exerts on diet. *D. eximius* adults and *L. berlandieri* exhibited a wider trophic niche (0.62 and 0.55, respectively) in wetter environments than Guadalcázar (Hernández-Salinas et al., 2018; Hernández-Austria, Luría-Manzano & Ramírez-Bautista, in press). Moreover, *A. debilis* adults and *S. multiplicata* non-adults showed a narrower trophic niche (0.15 and 0.1, respectively) in drier localities than Guadalcázar (Castañeda-Gaytán et al., 2006; Smith et al., 2011). These results suggest that trophic niche width contracts as aridity increases, in both dietary generalist and specialist species. Because anuran activity becomes more temporally limited in such conditions, interspecific competition for trophic resources may increase, leading to the contraction of trophic niche (Comas, Escoriza & Moreno-Rueda, 2014). Additionally, geographic variation in prey availability (which was not measured in any of these studies) could account for this pattern. Although pooling data from different years (as we did) has the risk of introducing some bias in our results because of climate variation, we have shown that there were no significant differences in interannual dietary composition in any species. Moreover, the Chihuahuan Desert is one of the arid regions with lowest interannual variation in rainfall (Van Etten, 2009).

In conclusion, the anuran community from the semi-arid region of Guadalcázar exhibited interspecific variation in dietary composition, with most species showing a narrow trophic niche width. Moreover, ontogenetic changes in dietary composition are widely present in this community, not being restricted to a certain family or species size; this supports the analysis of this phenomenon in any study of anuran community structure regarding diet (Hirai, 2002). Finally, by comparing our results with available data, it is evident that trophic niche width variation among populations is also widely present in the species analyzed. Therefore, clearly both intrinsic (ontogeny) and extrinsic (environment) factors influence dietary aspects of these anuran species.

## Supplemental Information

10.7717/peerj.7908/supp-1 Supplemental Information 1 Map of the study area. The state of San Luis Potosí, the municipality of Guadalcázar, and the five localities sampled are shown.

10.7717/peerj.7908/supp-2 Supplemental Information 2 Temperature and rainfall. Monthly temperature (circles) and rainfall (squares) based on a period of 30-year means (from 1981 to 2010) from the study area at Presa de Guadalupe, Guadalcázar, San Luis Potosí, Mexico (from CONAGUA-DGE, 2018).

10.7717/peerj.7908/supp-3 Supplemental Information 3 Costello’s modified representation of the feeding strategy of anurans studied. Costello’s modified (by Amundsen, Gabler & Staldvik, 1996) representation of feeding strategy by species and age class based on numeric (white circles) and volumetric (black circles) proportions. Prey categories with both values of *P*_*i*_ and FO lower than 0.2 are not shown (Salvidio et al., 2012). Abbreviations are the same as in Table S1, plus Ac, Acari; An, Anura; Ar, Araneae; Av, Aves; Coll, Collembola; De, Dermaptera; Di, Diplopoda; DiA, Diptera adults; DiL, Diptera larvae; Ep, Ephemeroptera; He, Hemiptera; Ho, Homoptera; Hym, Hymenoptera (others); Iso, Isoptera; LeL, Lepidoptera larvae; Odo, Odonata; Or, Orthoptera. For clarity, some prey categories are not labeled.

10.7717/peerj.7908/supp-4 Supplemental Information 4 Body size, and number and volume of each prey category consumed by each organism.

10.7717/peerj.7908/supp-5 Supplemental Information 5 Pairwise comparisons of diet between species. Pairwise comparisons of diet for numeric and volumetric proportions of prey categories between anuran species, showing the two prey that contributed most to the significant differences.

10.7717/peerj.7908/supp-6 Supplemental Information 6 Correlations between anuran size and prey types. Results of Spearman’s correlations between SVL and proportion of prey types that had a FO ≥ 30% in any of the age classes, in those anuran species that showed ontogenetic diet shifts according to Mantel tests.

## Additional Information and Declarations

### Field Study Permissions

The following information was supplied relating to field study approvals (i.e., approving body and any reference numbers): Specimens were collected under scientific permit SGPA/DGVS/01920/11 provided by SEMARNAT.

### Data Availability

The following information was supplied regarding data availability: The raw data and full specimen numbers are available in Data S1.
