# Supplementary figures and images for: Ontogenetic variation in the diet of the anuran community from a semi-arid environment in the southeastern Chihuahuan Desert
Source: PeerJ. 2019 Oct 18;7:e7908. doi: 10.7717/peerj.7908 (PMC6802674; doi:10.7717/peerj.7908)

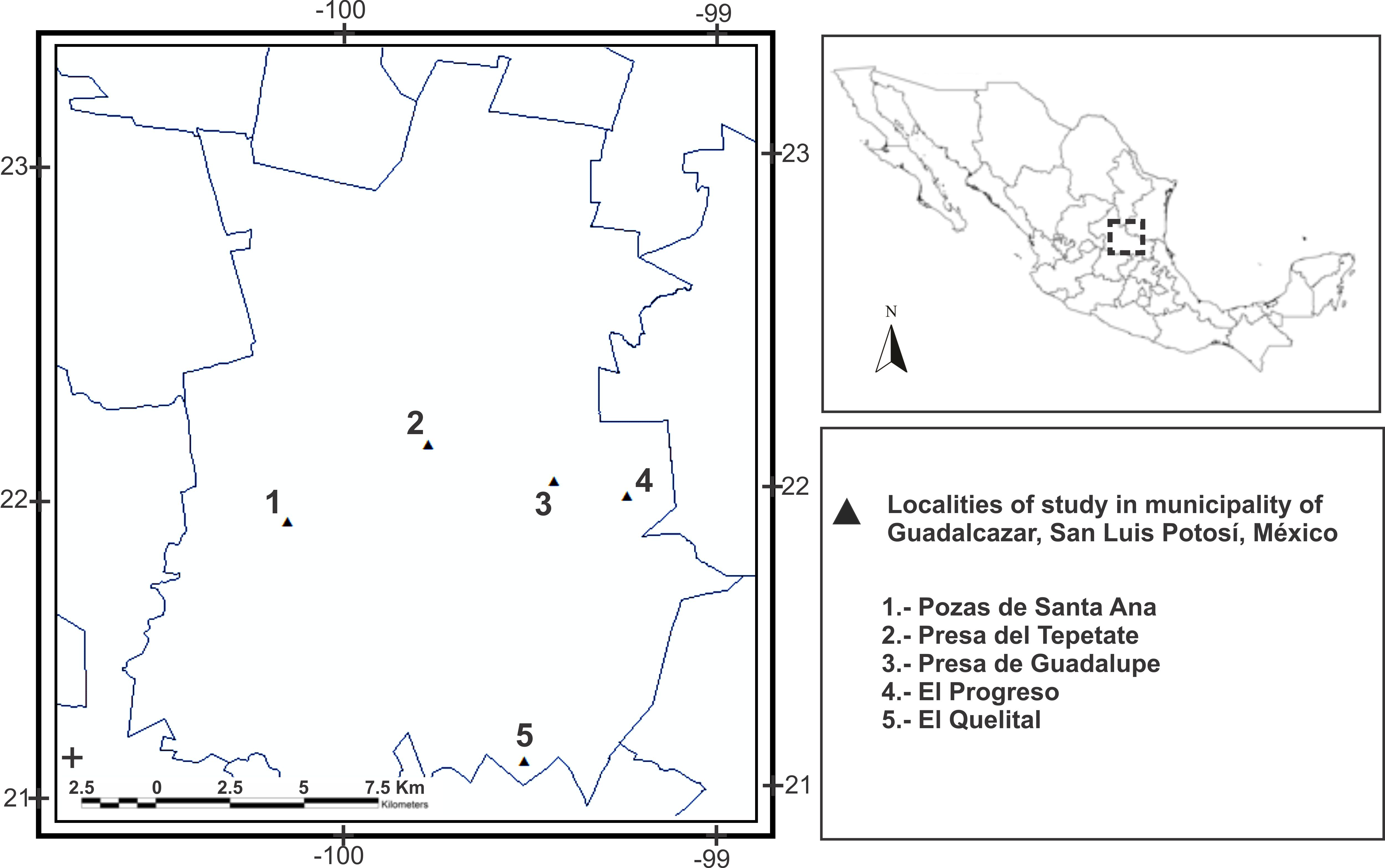

Supplement: Supplemental Information 1 — The state of San Luis Potosí, the municipality of Guadalcázar, and the five localities sampled are shown. [file peerj-07-7908-s001.jpg]

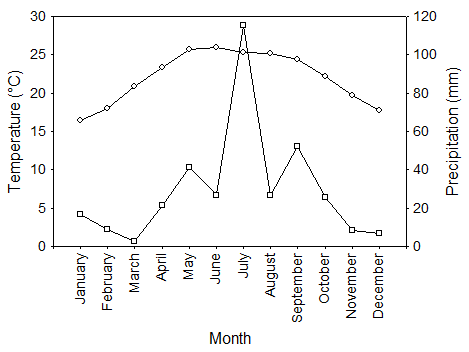

Supplement: Supplemental Information 2 — Monthly temperature (circles) and rainfall (squares) based on a period of 30-year means (from 1981 to 2010) from the study area at Presa de Guadalupe, Guadalcázar, San Luis Potosí, Mexico (from CONAGUA-DGE, 2018). [file peerj-07-7908-s002.png]

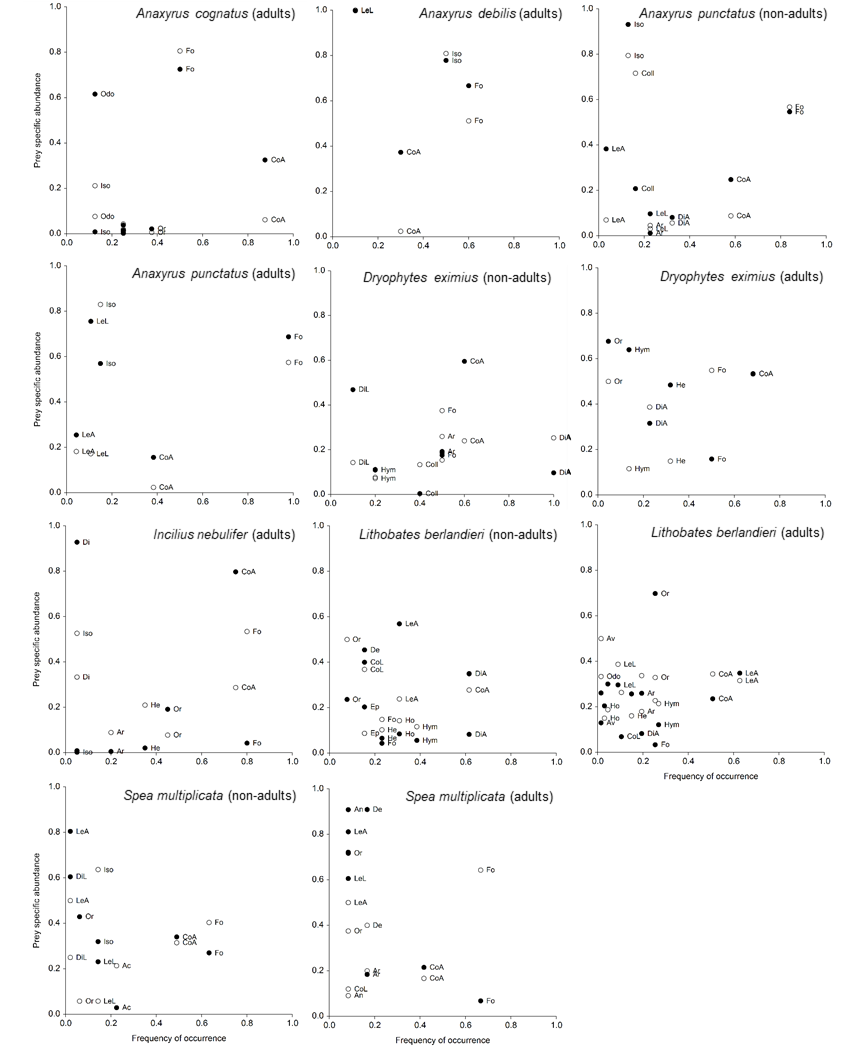

Supplement: Supplemental Information 3 — Costello’s modified (by Amundsen, Gabler & Staldvik, 1996) representation of feeding strategy by species and age class based on numeric (white circles) and volumetric (black circles) proportions. Prey categories with both values of Pi and FO lower than 0.2 are not shown (Salvidio et al., 2012). Abbreviations are the same as in Table S1, plus Ac, Acari; An, Anura; Ar, Araneae; Av, Aves; Coll, Collembola; De, Dermaptera; Di, Diplopoda; DiA, Diptera adults; DiL, Diptera larvae; Ep, Ephemeroptera; He, Hemiptera; Ho, Homoptera; Hym, Hymenoptera (others); Iso, Isoptera; LeL, Lepidoptera larvae; Odo, Odonata; Or, Orthoptera. For clarity, some prey categories are not labeled. [file peerj-07-7908-s003.png]
