# Supplementary material for: Ontogenetic variation in the diet of the anuran community from a semi-arid environment in the southeastern Chihuahuan Desert
Source: PeerJ. 2019 Oct 18;7:e7908. doi: 10.7717/peerj.7908 (PMC6802674; doi:10.7717/peerj.7908)
Supplement: Supplemental Information 6 — Results of Spearman’s correlations between SVL and proportion of prey types that had a FO ≥ 30% in any of the age classes, in those anuran species that showed ontogenetic diet shifts according to Mantel tests. [file peerj-07-7908-s006.docx]

|  | *A. punctatus* | | | | |  | *D. eximius* | | | | |  | *L. berlandieri* | | | | |
| --- | --- | --- | --- | --- | --- | --- | --- | --- | --- | --- | --- | --- | --- | --- | --- | --- | --- |
|  | Number | |  | Volume | |  | Number | |  | Volume | |  | Number | |  | Volume | |
| Prey category | *R_S_* | *P* |  | *R_S_* | *P* |  | *R_S_* | *P* |  | *R_S_* | *P* |  | *R_S_* | *P* |  | *R_S_* | *P* |
| Araneae | – | – |  | – | – |  | -0.418 | 0.017 |  | -0.419 | 0.017 |  | – | – |  | – | – |
| Coleoptera (A) | -0.252 | 0.026 |  | -0.231 | 0.042 |  | 0.399 | 0.024 |  | 0.329 | 0.066 |  | -0.178 | 0.114 |  | -0.237 | 0.034 |
| Collembola | – | – |  | – | – |  | -0.537 | 0.002 |  | -0.535 | 0.002 |  | – | – |  | – | – |
| Diptera (A) | -0.326 | 0.004 |  | -0.303 | 0.007 |  | -0.522 | 0.002 |  | -0.6 | <0.001 |  | -0.226 | 0.044 |  | -0.251 | 0.025 |
| Formicidae | 0.117 | 0.309 |  | 0.148 | 0.198 |  | 0.064 | 0.73 |  | -0.001 | 0.999 |  | – | – |  | – | – |
| Homoptera | – | – |  | – | – |  | -0.466 | 0.007 |  | -0.477 | 0.006 |  | -0.347 | 0.002 |  | -0.346 | 0.002 |
| Lepidoptera (A) | – | – |  | – | – |  | – | – |  | – | – |  | 0.367 | <0.001 |  | 0.334 | 0.003 |
